# Cleidocranial Dysplasia Causing Respiratory Distress in Neonates: A Case Report and Literature Review

**DOI:** 10.3389/fgene.2021.696685

**Published:** 2021-09-24

**Authors:** Ru Xue, Guoqing Zhang, Xiafang Chen, Xiuxia Ye

**Affiliations:** Department of Neonatology, Shanghai Children's Medical Center Affiliated to Shanghai Jiaotong University School of Medicine, Shanghai, China

**Keywords:** neonate, respiratory distress, *RUNX2*, mutation, cleidocranial dysplasia

## Abstract

Cleidocranial dysplasia (CCD; OMIM 119600) is a rare autosomal dominant skeletal dysplasia, which is mainly characterized by persistently open or delayed closure of fontanelle, patent skull sutures, abnormal clavicles, pectus excavatum, short stature, supernumerary teeth, and sinus and middle ear infections. It is caused by *Runt-related transcription factor 2* (*RUNX2; OMIM 600211*) mutations. Herein, we present a rare case of CCD with neonatal respiratory distress, who had abnormal midfacial features and wide fontanelle. Also, pectus excavatum was noted. He was transferred to our department, administered standard medical treatment, and discharged after 4 weeks. Therefore, we recommend the early suspicion and identification of this rare inherited disease to adequate treatment.

## Introduction

Cleidocranial dysplasia (CCD) is a rare autosomal dominant inherited disorder that was first identified by Marie and Sainton in 1898 (Marie and Sainton, 1898). It has an estimated incidence of 1/1000,000, with no gender or ethnic predilection (Ma et al., 2018). The disorder is caused by the runt-related gene *RUNX2*, which is essential for osteoblast differentiation and skeletal development (Wu et al., 2014). The pathogenic variants of *RUNX2* have a high penetrance and marked variability of expression, and some cases present with unusual signs and symptoms (Ramos et al., 2018). This report describes a premature newborn who suffered from respiratory distress after birth, requiring intensive care, as continuous positive airway pressure (CPAP), mechanical ventilation, and longer duration of antibiotic treatment. Physical examination revealed midfacial and cranial dysplasia, and chest deformity, palpation found the absence of parietal bones, wide bony sutures, and the anterior fontanelle reaching the nasion. Thoracic x-ray showed abnormal clavicles and ribs. Three-dimensional CT of the skull revealed delayed ossification, and wide sutures and fontanelles. Whole exome sequencing (WES) identified in the neonate a heterozygous pathogenic variant in *RUNX2* (c.568 C > T, p. Arg190Trp), allowing the diagnosis of CCD. This case demonstrates the importance of a timely diagnosis in children with unusual characteristics, in the presence of family history and clinical characteristics, which would allow adequate treatment.

## Case Presentation

**Figure 1:**
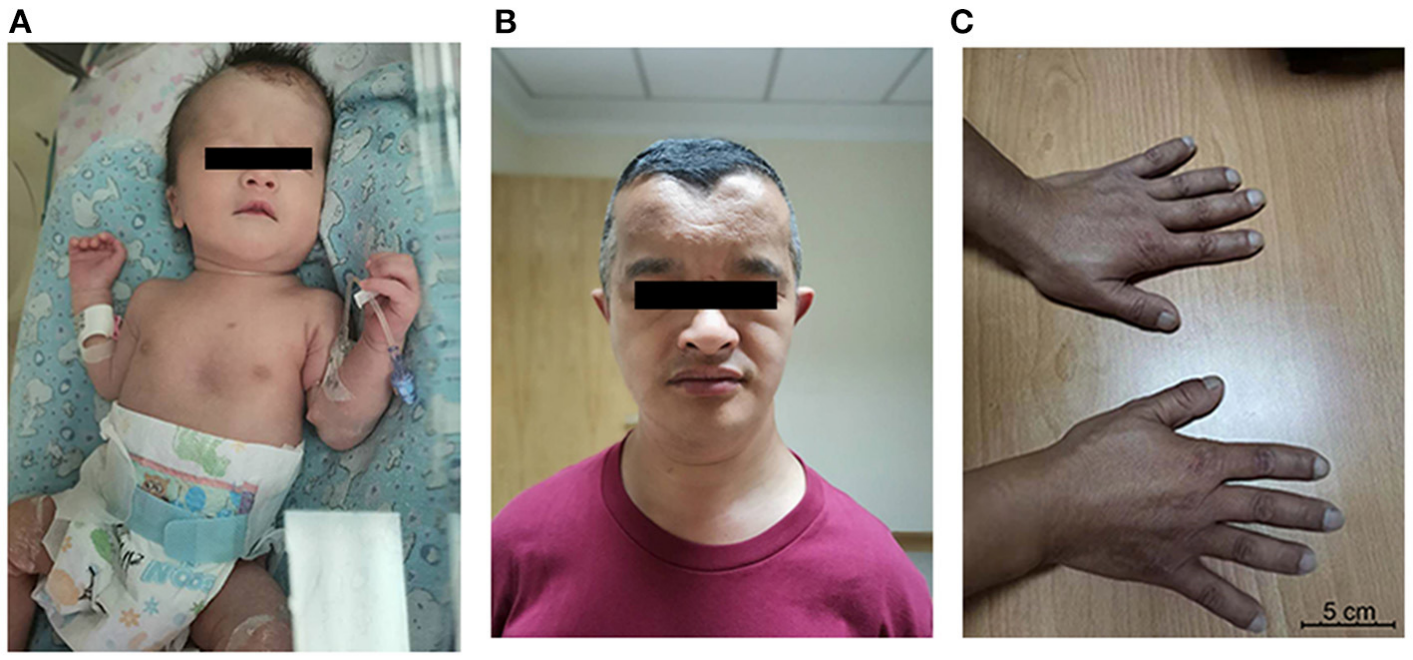
**(A)** Propositus: broad forehead with open metopic suture, pectus excavatum; **(B)** father: mediofrontal depression, ocular hypertelorism, malar hypoplasia; **(C)** father's hands: brachydactyly, brachytelephalangism, and broad thumbs.

A male infant, weighing 2,500 g, was born at 35 weeks' gestation by cesarean section delivery to a 41-year-old (six gravida, one para) mother who suffered from hypothyroidism and diabetes mellitus during pregnancy. The infant's Apgar scores were 9, 10, and 10 at 1, 5, and 10 min, respectively. His birth weight was 2,500 g (50th percentile), head circumference was 32 cm (50th percentile), and length was 46 cm (50th percentile). He was transferred to our department due to RDS. Physical examination showed rapid breathing, positive three concave sign, obvious pectus excavatum, abnormal midfacial features, and large fontanelle (Figure 1A). At cranial palpation, wide open fontanelles and sutures were noted.

Subsequent interviews with his family members revealed that his father is short (159 cm, below the 5th percentile) and has craniofacial dysmorphies such as frontal bossing with midfrontal depression, ocular hypertelorism, and supernumerary teeth. His shoulders are sloped and could be adducted toward the midline, and his clavicles are hypoplastic and fragmented. His hands show brachydactyly, brachytelephalangism, and broad thumbs (Figures 1B,C). He refers hearing disorders, and his intellect is normal. The baby's grandfather, who had passed away, had similar features.

**Figure 2:**
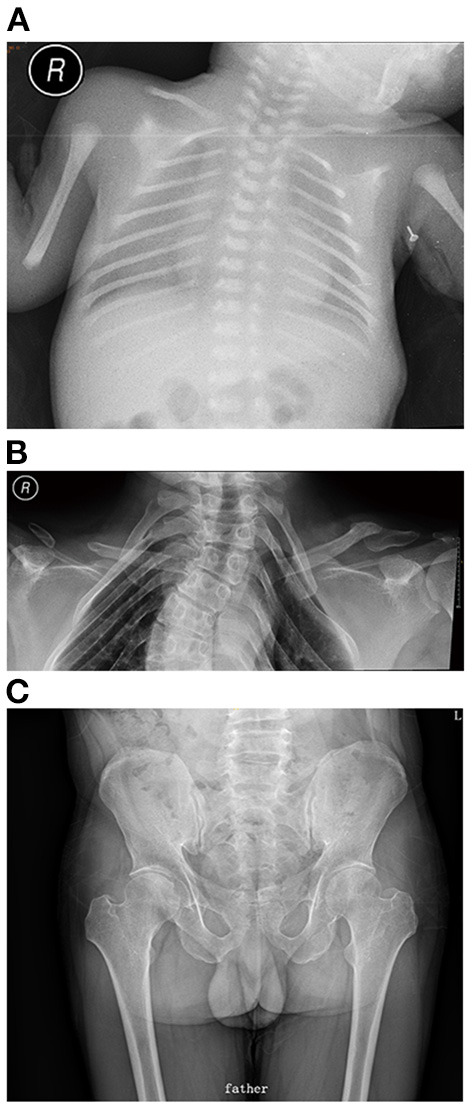
**(A)** Chest x-ray of the propositus: cone-shaped chest, clavicular hypoplasia, enlarged shoulder joint space. **(B)** Chest x-ray of father: cone-shaped chest, fragmented clavicles, scoliosis. **(C)** Pelvic x-ray of father: narrow pelvis, with short femoral necks, and short pubic rami.

**Figure 3:**
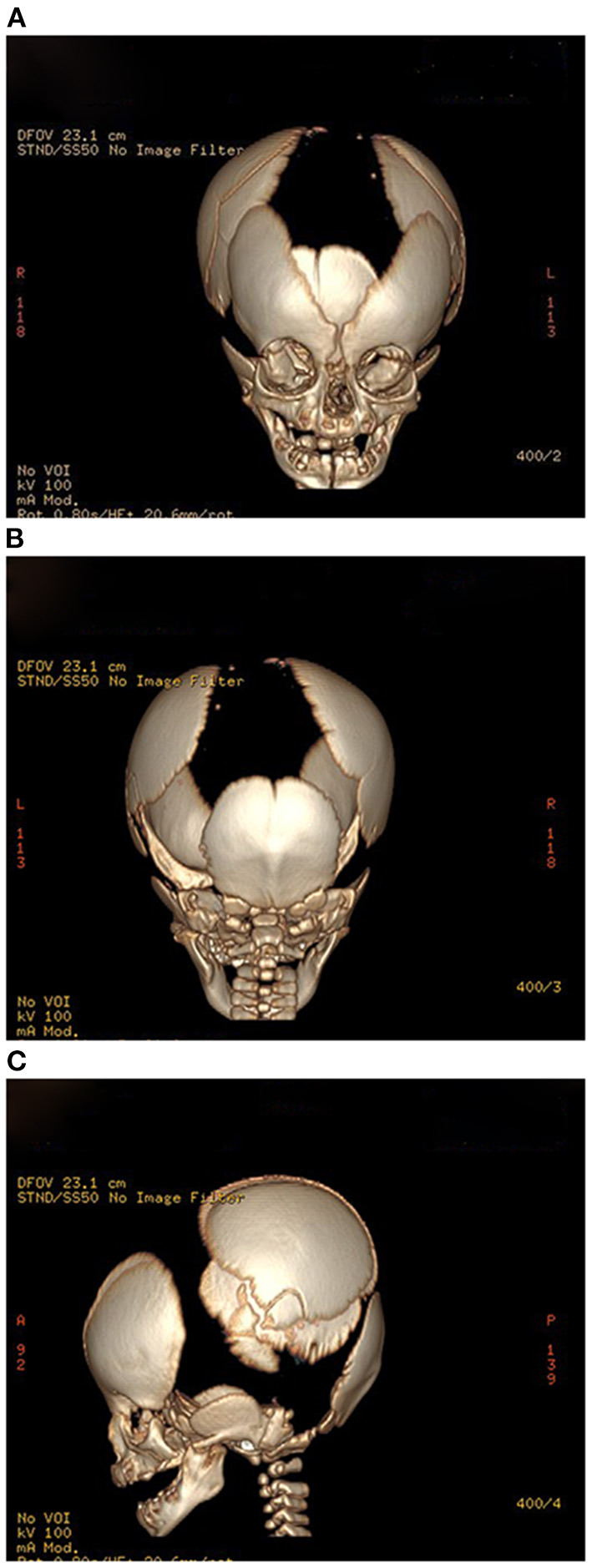
**(A–C)** Propositus skull CT, frontal, posterior, and lateral views: delayed ossification and wide-open sutures.

**Figure 4:**
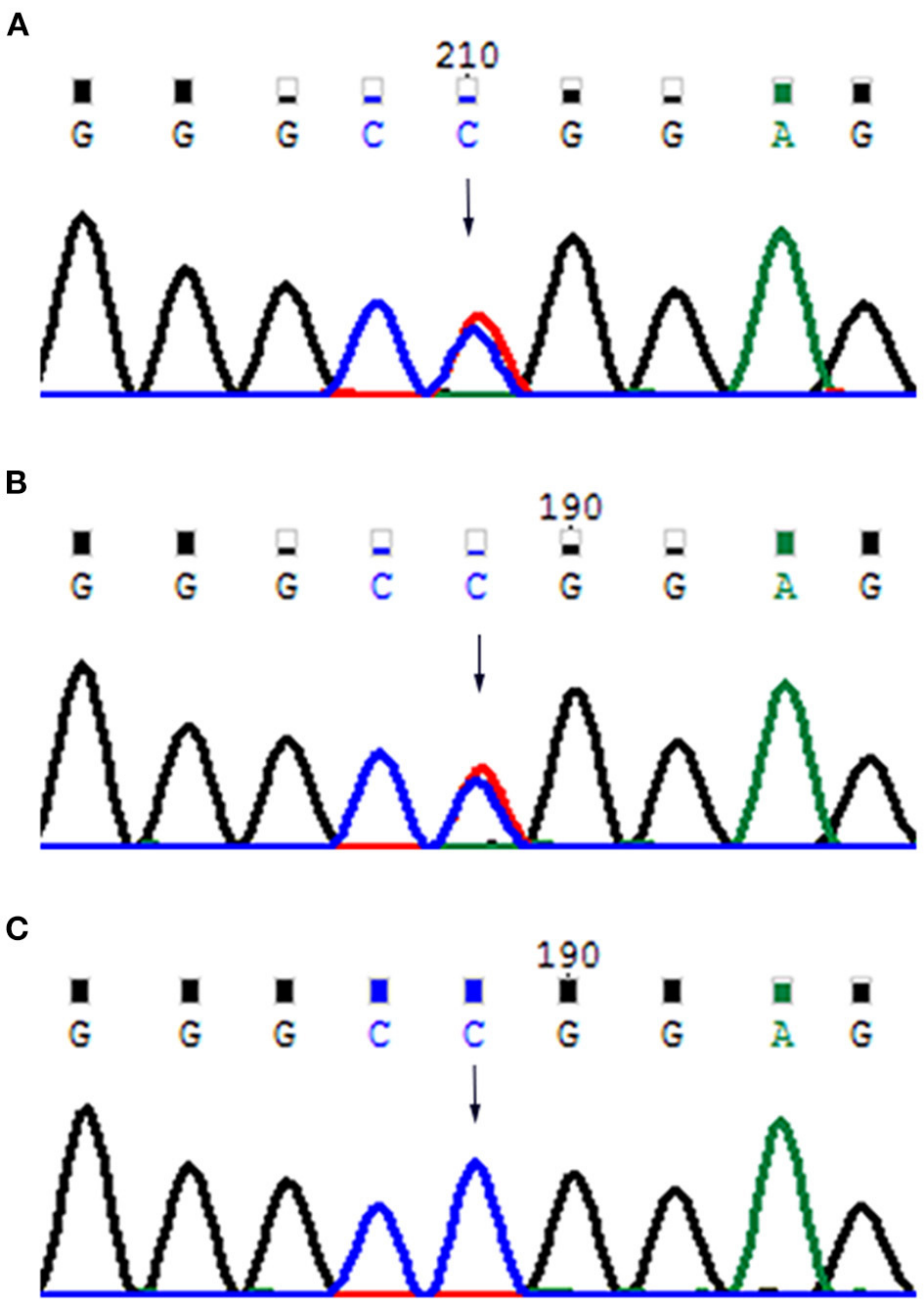
**(A)** Father's mutant *RUNX2* gene; **(B)** baby's mutant *RUNX2* gene; **(C)** mother's wild-type *RUNX2* gene.

The results of laboratory tests, including alkaline phosphatase, were not remarkable. Screening for hereditary metabolic disease was negative. Conventional chest radiography showed hypoplastic clavicles and cone-shaped chest, with enlarged shoulder joint space (Figure 2A). The radiology of his father showed short and fragmented clavicles, a cone-shaped chest, and scoliosis (Figure 2B); narrow pelvis, with short femoral necks and short pubic rami (Figure 2C). Three-dimensional CT of the baby showed large ossification defects in the skull (Figures 3A–C). Hip ultrasound showed dysplasia. Unfortunately, x-rays of the rest of the skeleton were not available. Based on the neonate's special facial characteristics, abnormal skeletal construction, radiography findings, and his family history, the diagnosis of CCD was confirmed. After obtaining informed consent from his parents, WES was performed. The results showed the existence of a missense mutation within *RUNX2* (c.568C>T, p. Arg190Trp), which was inherited from his father (Figure 4).

After standard medical treatment for respiratory distress syndrome (RDS), the child was discharged 4 weeks after birth. He was in a good state of health, although his pectus excavatum remained obvious along with minor tachypnea.

## Discussion

CCD, first introduced in 1898 by Marie-Sainton, and also known as Scheuthauer Marie-Sainton syndrome, is a rare autosomal dominant disorder with general retardation in bone ossification, apparently characterized by hypoplastic clavicles and various craniofacial abnormalities (Marie and Sainton, 1898). There are rare reports of possible autosomal recessive inheritance (Goodman et al., 1975). CCD spectrum disorder can be diagnosed prenatally. In case of a positive family history, prenatal ultrasound can be used to identify abnormal skull ossification combined with short clavicles in a fetus, to establish an early diagnosis (Hassan et al., 1997; Hermann et al., 2009). However, our patient was not diagnosed during antenatal care.

*RUNX2* plays a pivotal role in osteoblast differentiation. It was previously named polyomavirus enhancer binding protein 2A or core binding factor A1 (Mundlos et al., 1995). It has been mapped to chromosome 6p21 (Mundlos et al., 1997). *RUNX2* is a major regulatory transcription factor that controls the differentiation of precursor cells into osteoblasts, and is essential for both membranous and endochondral development bone formation. It is called the “master gene” of bone development. Approximately 60–70% of the reported cases of CCD carry pathogenic mutations of *RUNX2* (Machol et al., 2020). The c.568C>T (p. Arg190Trp) missense mutation identified in the present study has been previously reported (Jaruga et al., 2016). However, the underlying mechanisms of *RUNX2* in osteogenic differentiation remain less understood. A recent study indicated that Y1 receptor deficiency stimulates the Camp/PKA/CREB pathway leading to activation of *RUNX2*, which enhances osteogenic differentiation (Yu et al., 2020). Hence, the disease is characterized by general skeletal dysplasia.

Some previously reported cases were diagnosed in the neonatal period, but respiratory distress has rarely been reported. Our patient was not at high risk for RDS, his gestational age was 35 weeks, and he did not present typical radiographic images of respiratory distress. However, he required prolonged ventilatory support, which could be related to the restrictive thoracic deformity.

Similarly, in another report, a preterm newborn with RDS required CPAP ventilation after birth. The baby had small skull bones, a large fontanelle, and wide sutures, leading to the diagnosis of CCD. Symptoms of respiratory distress were associated with chest deformity (Ringe et al., 2010). Chitayat et al. reported a girl diagnosed with CCD at 5 months of age, with a positive family history. She had respiratory distress, due to a narrow chest and required tube feeding for 5 months due to swallowing difficulties (Chitayat et al., 1992). These two cases provide evidence of CCD causing respiratory distress; therefore, healthcare professionals should be aware of this possible complication related to thoracic deformity.

Although respiratory problems have rarely been reported in newborns with CCD, thoracic deformity has been frequently described in these patients. Seven of 14 CCD patients aged 1 week to 49 years, who had a radiological examination, had positive radiological findings, including pectus carinatum or excavatum, and required the attention of orthopedic surgeons (Jirapinyo et al., 2020). Also, another report describes a child with pectus excavatum who required the Nuss procedure (Takagi et al., 2020).

Hypoplastic chest can cause respiratory failure (Fauré and Nahon, 1969); however, other complications should be considered when neonates with CCD present with respiratory distress. Shohei et al. reported a full-term newborn with a family history of CCD. Shortly after the vaginal delivery, she developed apnea, vomiting, and bradycardia. CT of the skull and chest radiograph showed the characteristics of CCD and revealed a subdural hematoma of the posterior fossa (PFSDH). PFSDH was thought to be the result of forces produced during vaginal delivery on a more fragile skull, resulting in respiratory compromise (Nagasaka et al., 2020).

The differential diagnosis of CCD includes other skeletal dysplasia, such as picchondisostosis, mandibuloacral dysplasia, hypophosphatasia, osteogenesis imperfecta, and Yunis–Varon syndrome. Other conditions such as congenital pseudoarthrosis of the clavicle, hypothyroidism, and chromosomal abnormalities should also be considered (Machol et al., 2020).

Previously reported patients from China were older children or adults with classic skeletal abnormalities (Shen et al., 2009; Wang et al., 2013). To our knowledge, this is the first newborn reported with CCD and RDS in China. In this case, the family history and radiological findings allowed the diagnosis of CCD; in addition, we identified a previously reported pathogenic variant of the *RUNX2* gene. In cases of children with a positive family history, early detection of this condition, even prenatally, would prevent complications and adapt the instituted treatments.

## Conclusion

This case provides further evidence about the possibility of respiratory problems in newborns with CCD. Early identification of this condition, especially in cases with a positive family history, could help plan a safe delivery and adjust respiratory treatment.

## Data Availability Statement

The original contributions presented in the study are included in the article/supplementary materials, further inquiries can be directed to the corresponding author/s.

## Ethics Statement

Written informed consent was obtained from the individual(s) for the publication of any potentially identifiable images or data included in this article.

## Author Contributions

RX and GZ conceptualized and designed the study, drafted the initial article, and reviewed the article. XC and XY designed the data collection instruments, coordinated and supervised data collection, and critically reviewed the article. All authors approved the final article as submitted and agree to be accountable for the content of the work.

## Conflict of Interest

The authors declare that the research was conducted in the absence of any commercial or financial relationships that could be construed as a potential conflict of interest.

## Publisher's Note

All claims expressed in this article are solely those of the authors and do not necessarily represent those of their affiliated organizations, or those of the publisher, the editors and the reviewers. Any product that may be evaluated in this article, or claim that may be made by its manufacturer, is not guaranteed or endorsed by the publisher.
